# Social isolation and 59 common health conditions: insights from observational and genetics analyses

**DOI:** 10.1186/s12916-026-04784-1

**Published:** 2026-03-23

**Authors:** Sizhi Ai, Qiqi Wu, Yu Nie, Yiliang Ou, Yu He, Puiyin Li, Qi Yong H. Ai, Hongliang Feng, Yaping Liu, Tong Luo, Jihui Zhang, Yannis Yan Liang

**Affiliations:** 1 Center for Sleep and Circadian Medicine, The Affiliated Brain Hospital, Guangzhou Medical University, Guangzhou, Guangdong 510370 China; 2 Key Laboratory of Sleep and Biological Rhythms of Guangdong Province, Guangzhou Medical University, Guangzhou, Guangdong 510260 China; 3 Key Laboratory of Neurogenetics and Channelopathies of Guangdong Province and the Ministry of Education of China, Guangzhou Medical University, Guangzhou, Guangdong 510260 China; 4 Institute of Psycho-Neuroscience, The Affiliated Brain Hospital, Guangzhou Medical University, Guangzhou, Guangdong 510370 China; 5 Renal Division, Peking University First Hospital, Beijing, 100032 China; 6 Institute of Nephrology, Peking University, Beijing, 100032 China; 7 Department of Psychology, The Chinese University of Hong Kong, Hong Kong SAR, China; 8 Department of Imaging and Interventional Radiology, Faculty of Medicine, The Chinese University of Hong Kong, Hong Kong SAR, China

**Keywords:** Social isolation, Multiple health conditions, UK Biobank, Mendelian randomization

## Abstract

**Background:**

The impacts of social isolation on diverse health conditions and how it contributes to health risks remain unclear. We aimed to investigate the associations of social isolation with 59 health conditions among older adults.

**Methods:**

Participants from the UK Biobank without baseline diagnosis of the included diseases were selected. Social isolation was assessed with three questions. The 59 health conditions included all-cause mortality, 5 cause-specific mortalities, and 53 diseases. We used an instrumental variable from multivariable common factor GWAS in Mendelian randomization (MR) to explore causal links of social isolation with diseases. Omics analyses were conducted to assess the roles of Olink plasma proteins and metabolomics, and PERM was calculated to evaluate the influence of other factors.

**Results:**

A total of 489,741 individuals [266,706 (54.5%) women; mean age 56.5 years (SD 8.1)] were included. During a median follow-up of 12.5 years, social isolation was uncorrelated with the majority of 59 health conditions. Significantly, it was associated with increased risks of all-cause [adjusted HR (aHR) 1.28, 95% CI 1.25–1.32], 5 cause-specific mortalities (aHR range, 1.18–1.38), and 11 specific diseases (aHR range, 1.08–1.17). Living alone was the strongest item of isolation in predicting mortality (aHR range, 1.18–1.45) and selected diseases. MR analyses offered little evidence to support a causal link between social isolation and these diseases. The proteins involved in these associations are predominantly related to “response to stimulus”. Proteomic signatures (PERM, 36%–49%), health behaviours (32%–59%), and socioeconomic factors (22%–42%) were the main explanatory factors linking social isolation to 8 health outcomes.

**Conclusions:**

Social isolation is associated with elevated risks of 17 out of the 59 examined adverse health outcomes, predominantly mortality-related conditions; however, MR analyses indicate an absence of evidence supporting causality for these associations.

**Supplementary Information:**

The online version contains supplementary material available at 10.1186/s12916-026-04784-1.

## Background

Social connection constitutes a fundamental need of human beings [1]. Social isolation, characterized by an inadequate quantity of social connection, is distinct from loneliness (a painful feeling arising from poor quality of social connection) [2]. A climbing number of older adults are experiencing social isolation [3], potentially due to factors such as retirement, the loss of a spouse, or children moving away [4]. Meanwhile, older adults often experience multiple illnesses or comorbidities [5]. However, most existing studies concerning social isolation have narrowly concentrated on single disease conditions within the middle-aged population [6–8]. Consequently, there is a scarcity of evidence investigating the associations between social isolation and a broad spectrum of health conditions among older adults.

A hot debate continues on whether the association between social isolation and diseases is causal. Growing evidence supports robust associations between social isolation and heightened risks for various diseases, like cardiovascular disease [9, 10], depression [11], and dementia [12]. Yet, some studies [13], including ours [14], suggest that living alone, a key item of social isolation, only increases the risk of mortality and fatal diseases, but not non-fatal conditions. These findings provide limited evidence to support a causal relationship. The discrepancies in observations may arise from reverse causation and residual confounding of the observational design. Instead, Mendelian randomization (MR) offers a promising solution to these limitations, providing genetically causal evidence [15]. Several pioneering MR studies have revealed a potentially causal link from social isolation to diabetes [16] and depressive symptoms [17]. However, it remains unclear whether this causal evidence extends to a wide range of health conditions.

It is intriguing to explore potential biological correlates linking social isolation to health conditions. Prior research has shown that social isolation influences the risk of cardiovascular diseases and mortality by impacting various factors, such as health behaviours, psychological factors, and comorbidities [18, 19]. Beyond these explanatory factors, characterizing molecular signatures that are associated with social isolation and disease conditions could pave the way for more precise prevention and intervention strategies. Social isolation has been suggested to modulate circulating proteins and metabolic processes [20]. A recent experimental study suggests that social isolation may promote atherosclerosis via oxytocin-related pathways [21]. However, evidence regarding proteomic associations of social isolation with a wide range of health conditions is rare. Additionally, the specific molecular pathways that are associated with these relationships are yet to be fully understood.

Taking advantage of a large sample of individual-level data from the UK Biobank, the present study primarily aimed to identify the associations between social isolation and the occurrence of 59 health conditions, encompassing 53 individual diseases across 14 disease categories, all-cause mortality and 5 cause-specific mortality conditions. Secondly, to ascertain whether these outcome-wide associations are causal, we initially investigated whether living alone was most strongly associated with severe physical and mental health conditions as compared to the other two items. Using MR analyses, we further examined causality between social isolation and multiple health conditions. Finally, by integrating behavioural, physical, and proteomic data, we investigated the potential explanatory factors underlying the observed associations between social isolation and each identified health condition.

## Methods

### Study design and participants

**Fig. 1:**
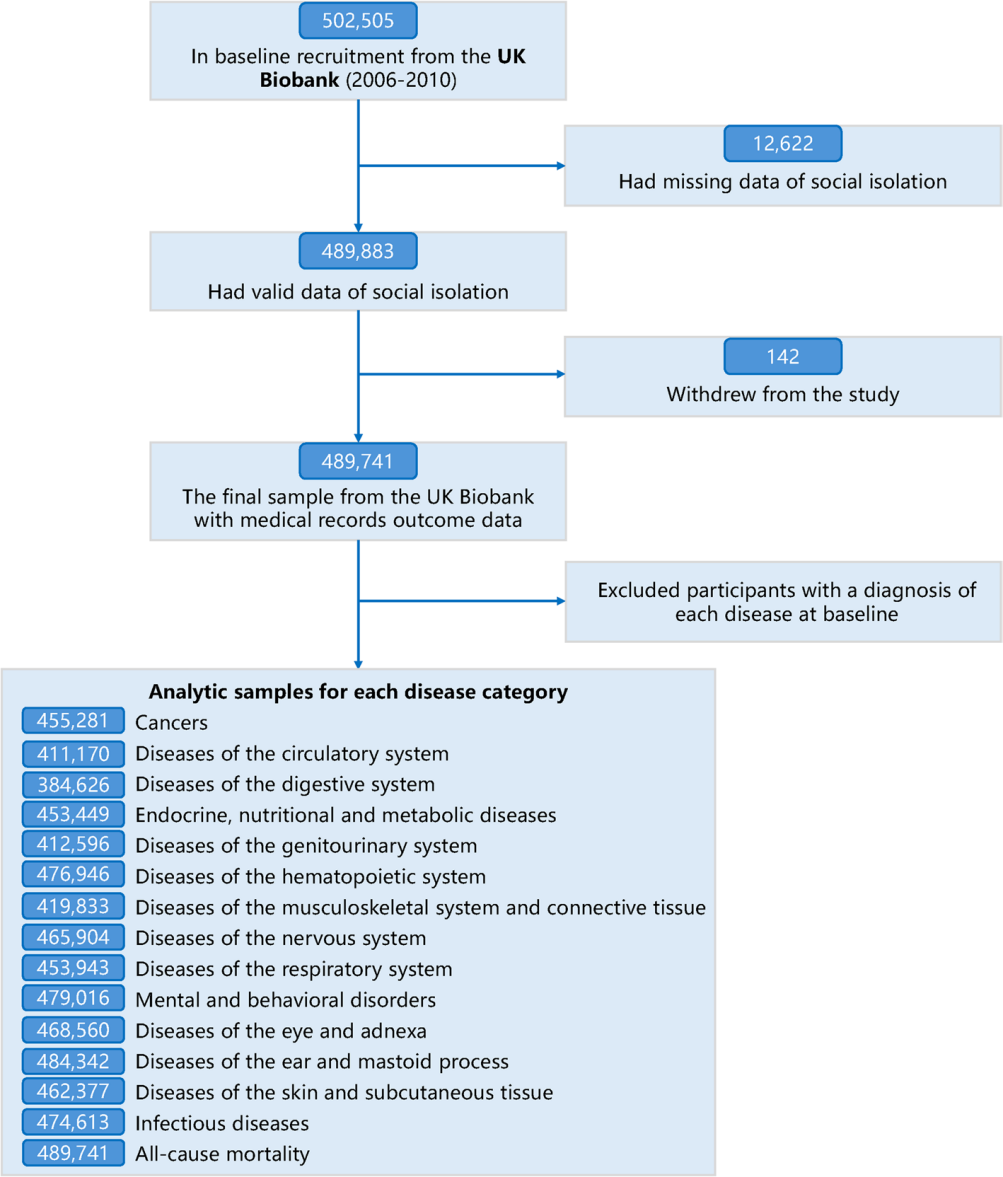
Study profile. The study included 489,741 participants with valid and complete answers about social isolation. We excluded participants with a diagnosis of each disease at baseline, respectively. The sample of each disease category was presented in the “Analytic samples for each disease category”

Data for this study were sourced from the UK Biobank, a large-scale prospective cohort encompassing over 500,000 participants aged 40 to 70 years, recruited between 2006 and 2010 [22]. Participant data include genome-wide genotypes, electronic health record linkage, blood biomarkers, and various other phenotypic assessments. Participants provided written informed consent. The study received approval from the National Health Service National Research Ethics Service (11/NW/0382). For the current analysis, a total of 489,741 participants were included (Fig. 1 and Additional file 1: Methods S1).

### Exposures

In the UK Biobank, the social isolation scale was measured using three questions [9, 18]: (1) “Including yourself, how many people are living together in your household?” (1 point = “one”; 0 point = more than one); (2) “How often do you visit friends or family or have them visit you?” (0 point = “Almost daily”, “2–4 times a week”, or “About once a week”; 1 point = “About once a month”, “Once every few months”, “Never or almost never”, or “No friends/family outside household”); (3) “Which of the following leisure/social activities do you engage in once a week or more often?” (1 point = “None of the above”; 0 point = “Sports club or gym”, “Pub or social club”, “Religious group”, “Adult education class”, or “Other group activity”). The social isolation scale was calculated by summing the scores of these three questions, resulting in a range of 0–3. We further classified participants as socially isolated (isolation scale ≥ 2) or not isolated (isolation scale < 2) [19, 23, 24]. Additionally, “living alone” was assessed using the first question, “little contact with friends or family” was evaluated based on the second question, and “fewer leisure/social activities” was identified through the third question. Details of the scoring methods are listed in Additional file 1: Methods S2.

### Outcomes

The primary outcomes for this study were the incidence of 53 individual diseases across 14 disease categories and 5 disease-specific mortalities according to the International Classification of Diseases-10th Revision (ICD-10) codes (Additional file 2: Table S1). We used the unique identification numbers assigned to all UK Biobank participants to link them to hospital admission data and death registry records. Participants were followed up until the occurrence of the first event, death, or cessation, whichever came first. The 14 disease categories were then subdivided into fatal and non-fatal subtypes. Events for participants who died without a prior hospital admission or on the first day of their hospital admission were classified as fatal subtypes [9, 14].

### Covariates

The potential covariates of our analysis included the following: age (continuous, years), sex (female/male), ethnicity (white/others), assessment centres (England/Scotland/ Wales), Townsend Deprivation Index, education level (college or university degree/non-college or university degree), smoking status (never/previous/current), alcohol intake frequency (not current/two or less times a week/three or more times a week), body mass index (BMI) (continuous, kg/m2), physical activity (continuous, minutes/week), and loneliness status (loneliness/no loneliness). Detailed information is provided in the Additional file 1: Methods S3 [25].

### Mendelian randomization

To investigate the potential causal associations between social isolation and disease outcomes, we conducted meta-analyses of bidirectional two-sample MR studies using summary-level data from genome-wide association studies (GWAS). Detailed information on the GWAS datasets for social isolation and the disease outcomes is provided in Additional file 2: Tables S2-S8 and Additional file 1: Methods S4 [16, 17, 26].

First, we performed a latent factor GWAS of social isolation, as the resulting instruments were used in the main MR analyses. Specifically, we jointly modelled the trait liability of “number in household”, “frequency of friend/family visits”, and “leisure/social activities: none of the above” using a common factor model implemented in genomic structural equation modelling. Before running the multivariable GWAS, alleles were aligned across the three traits using the HAPMAP3 reference panel. Single nucleotide polymorphisms (SNPs) were retained if they met the quality control criteria of minor allele frequency > 0.01 and INFO > 0.9. Using the 1000G European reference panel, we then estimate the linkage disequilibrium score regression (LDSC) for the three social isolation-related traits. Subsequently, the LDSC outputs and trait-specific summary statistics were combined to perform the multivariable common factor GWAS. The regression coefficients of “number in household” and “frequency of friend/family visits” were reversed so that higher values reflected greater social isolation rather than greater social contact [27].

Second, in the bidirectional two-sample MR studies, we focused on 9 out of 53 diseases that remained significant after Bonferroni correction in the Cox models and for which appropriate GWAS data were available. The selection criteria for genetic instruments in our study were as follows: (1) genome-wide significance (*P* < 5 × 10^−6^); and (2) linkage disequilibrium among SNPs associated with each exposure was calculated using the PLINK clumping method, based on the 1000 Genomes European panel. SNPs with *r*^2^ > 0.001 and a clumping distance < 10,000 kilobases (kb) were excluded. After applying these criteria, 13 SNPs were retained as valid genetic instruments for social isolation. Subsequently, we excluded SNPs associated with the disease outcomes (*P* < 0.05) to ensure that the instruments influenced the outcomes only through social isolation. The *F*-statistics of all SNPs were calculated to assess instrument strength, with values < 10 indicating potential weak instrumental variables. The random-effects multiplicative inverse variance-weighted (MRE-IVW) method was used to estimate the causal associations between social isolation and disease conditions [26]. Meta-analysis of the MR estimates from multiple outcome datasets was subsequently performed to enhance the robustness of the causal inference. Additionally, several MR sensitivity analyses, including weighted median [28] and ME-Egger [29] methods, were conducted to further assess the robustness of the results. The MR pleiotropy Residual Sum and Outlier (MR-PRESSO) [30] and Cochran’s *Q* test were also applied to evaluate horizontal pleiotropy and heterogeneity across SNPs’ estimates.

Finally, as additional sensitivity analyses, we conducted meta-analyses of bidirectional two-sample MR studies to examine the associations between each of the three traits of social isolation and the disease conditions that remained significant after Bonferroni correction in the Cox models. Moreover, the genetic variants associated with social isolation behaviour identified by Socrates et al. [17] were used as instruments for MR sensitivity analyses to further assess the robustness of the findings. Detailed information on these MR analyses is provided in Additional file 1: Methods S4 [16, 17].

### Proteomic and metabolomic measurements

Baseline biological samples in the UK Biobank were collected between 2006 and 2010. The UK Biobank Pharma Proteomics Project (UKB-PPP) consortium employed the Olink Explore 3072 Proximity Extension Assay to perform proteomic profiling on baseline blood plasma samples from 53,026 participants between April 2021 and February 2022 [20, 31]. NMR metabolomics data were also obtained from UK Biobank (category 220), generated by Nightingale Health using high-throughput NMR spectroscopy on EDTA plasma samples in three phases between June 2019 and November 2023, covering 251 metabolic markers across multiple pathways. All participants provided informed consent. In our analysis, a total of 2923 proteins and 251 metabolomic markers were included.

### Statistical analyses

Descriptive statistics for baseline characteristics of participants are presented as proportions for categorical variables and mean ± standard deviation (SD) or median [interquartile range (IQR)] for continuous variables. Missing data were addressed using multiple imputation with the “mice” R package to minimize potential inferential bias. Cox proportional hazards models, our primary analysis method, were employed to calculate hazard ratio (HR) and 95% confidence interval (CI) to evaluate the associations between social isolation and its items with the incidence of multiple diseases. The multivariate Cox models were adjusted for age, sex, ethnicity, assessment centres, Townsend Deprivation Index, education level, smoking status, alcohol intake frequency, BMI, physical activity, and loneliness status. Incident rates (cases per 1000 person-years) of outcome events were calculated [2, 32].

Several sensitivity analyses were performed to assess the robustness of the primary results. First, analyses were repeated using only complete cases by excluding participants with missing covariate data. Second, deaths or first events occurring within the initial 2 years were excluded. Third, the Fine-Gray subdistribution hazard model was applied to account for the competing risk of death. Fourth, propensity score matching (PSM) was performed to balance baseline covariates between social isolation groups, with details provided in Additional file 1: Methods S5 [33]. Fifth, to identify potential residual or unmeasured confounding, a negative control outcome (NCO) analysis was conducted using nonsuppurative otitis media, for which no evidence supports an association with social isolation. Detailed information on the NCO analyses is provided in Additional file 1: Methods S6 [34, 35]. Finally, inverse probability weighting (IPW) was applied to address selection bias inherent to the UK Biobank cohort. Details of the IPW procedure are provided in Additional file 1: Methods S7 [36, 37]. Additionally, subgroup analyses were conducted by stratifying the sample according to age (< 60 years or ≥ 60 years), sex (male or female), and loneliness status (loneliness or no loneliness). Interaction *P* values between social isolation, multiple diseases, and stratification variables were used to assess statistical significance.

To investigate the potential explanatory roles of proteomics and metabolomics in the associations of social isolation with multiple diseases and mortality, we performed exploratory observational analyses using individual protein and metabolite measurements. Proteomic and metabolomic analyses were conducted under two predefined analytical frameworks informed by the Mendelian randomization findings (see Additional file 1: Figure S1). First, multivariable-adjusted Cox proportional hazards regression models were employed to calculate HRs and 95% CIs for the relationships of individual omics signatures with risks of multiple diseases and mortality. Next, the associations between the significant omics signatures identified earlier and social isolation were assessed using multivariable-adjusted linear regression models [38]. The models were adjusted for age, sex, ethnicity, assessment centres, Townsend Deprivation Index, education level, smoking status, alcohol intake frequency, BMI, physical activity, and loneliness status. Subsequently, mediation analysis was performed on the significant omics signatures [39, 40]. Enrichment analysis was performed on the proteins with significant mediating roles, and detailed information is available in the Additional file 1: Methods S8 [20]. Finally, machine learning was applied to the omics signatures that showed significance in the previous analyses. An eXtreme Gradient Boosting (XGBoost) model was trained to predict the occurrence of specific diseases, and the top 15 proteins or metabolic markers with the highest predictive values were identified using Shapley Additive Explanations (SHAP) Details of the machine learning analyses are provided in Additional file 1: Methods S9 [41, 42]. Among these, the top 5 with the highest predictive abilities were selected, and percentage of excess risk mediated (PERM) values were calculated [20].

The PERM was calculated to evaluate the extent to which various explanatory factors accounted for the associations between social isolation and multiple diseases. The PERM was calculated using the following formula [19, 20]:

$$\mathrm{PERM} = \left[ \frac{\text{HR (adjusting for age, sex, ethinicity and assessment centre)} - \text{HR (adjusting for age, sex, ethinicity, assessment centre and explanatory factors)}}{\text{HR (adjusting for age, sex, ethinicity and assessment centre)} - 1} \right] \times 100\%$$

Although the mediation analysis and PERM were primarily aimed at identifying mediators along the pathways from exposure to outcome, the identified biomarkers may also act as confounders or colliders in the context of exposure and outcome (Additional file 1: Figure S1).

All models included social isolation as the exposure and covariates were added as follows: Model 1 included age, sex, ethnicity, and assessment centres. Subsequent models incorporated additional explanatory factors: socioeconomic factors (education level, Townsend Deprivation Index, and employment); health behaviours (health diet score, smoking status, alcohol intake frequency, sleep duration, and physical activity); baseline depressive symptoms (depression scores measured by Patient Health Questionnaire-2); inflammatory factors (leukocytes, platelets, platelet crit, lymphocytes, monocytes, neutrophils, eosinophils, basophils, C-reactive protein); comorbidities (history of long-standing illness, use of cholesterol-lowering medication, and use of antihypertensive medication); proteomics signatures (the top 5 proteins with the highest predictive value for each disease); and metabolomics signatures (the top 5 metabolic indicators with the highest predictive value for each disease).

Most statistical analyses were performed using R software (version 4.3.1), except for machine learning, which was conducted using Python (version 3.8). We also performed multiple comparisons using the Bonferroni correction, and a *P* < 0.05/number of comparisons was considered to indicate statistical significance. Specific R packages and their versions included: the mice package (v.3.16.0) for data imputation; the survival package (v.3.5.7) for performing Cox proportional hazards regression; the MatchIt package (v.4.5.5) for PSM; the ipw package (v.1.2.1.1) for IPW; the TwoSampleMR package (v.0.6.3) for conducting two-sample Mendelian randomization analysis; the MRPRESSO package (v.1.0) for performing MR-PRESSO analysis; the meta package (v.7.0.0) for meta-analysis; and the ggplot2 package (v.3.5.0) and forestplot package (v.1.1.2) for data visualization.

### Patient and public involvement

Patients and the public were not involved in the design, conduct, reporting, or dissemination plans of our research.

## Results

### Baseline characteristics

The baseline characteristics of the entire cohort are presented in Table 1. The main analysis included 489,741 participants from the UK Biobank with available medical condition data. The mean age of the cohort was 56.5 years (SD 8.1 years), with 266,706 participants (54.5%) being women (Table 1). A total of 70,998 participants (14.5%) were classified as socially isolated. Compared with non-isolated individuals, those who were socially isolated were more likely to be male, obese, currently employed, frequent smokers, and to report higher levels of loneliness. After PSM, no major imbalances in baseline characteristics were observed (Additional file 2: Table S9).

**Table 1:** Baseline characteristics of participants in the UK Biobank by social isolation status

Characteristics	Total (*N* = 489,741)	Not isolated (*N* = 418,743)	Socially isolated (*N* = 70,998)
Age (years) (mean (SD))	56.5 (8.1)	56.6 (8.1)	56.1 (7.9)
Sex, female, *n* (%)	266,706 (54.5%)	231,540 (55.3%)	35,166 (49.5%)
Ethnicity, White, *n* (%)	464,452 (94.8%)	398,558 (95.2%)	65,894 (92.8%)
Assessment center, *n* (%)			
England	433,862 (88.6%)	370,485 (88.5%)	63,377 (89.3%)
Scotland	35,345 (7.2%)	30,346 (7.2%)	4999 (7.0%)
Wales	20,534 (4.2%)	17,912 (4.3%)	2622 (3.7%)
Townsend deprivation index^a^	− 2.2 [− 3.7; 0.5]	− 2.3 [− 3.7; 0.2]	− 1.1 [− 3.1; 2.1]
College or university degree, *n* (%)	160,382 (32.7%)	136,832 (32.7%)	23,550 (33.2%)
Currently employed, *n* (%)	284,194 (58.0%)	240,805 (57.5%)	43,389 (61.1%)
Smoking status, *n* (%)			
Never	268,265 (54.8%)	232,487 (55.5%)	35,778 (50.4%)
Previous	170,112 (34.7%)	146,464 (35.0%)	23,648 (33.3%)
Current	51,364 (10.5%)	39,792 (9.5%)	11,572 (16.3%)
Alcohol intake frequency, *n* (%)			
Not current	38,823 (7.9%)	30,317 (7.2%)	8506 (12.0%)
Two or less times a week	237,310 (48.5%)	199,974 (47.8%)	37,336 (52.6%)
Three or more times a week	213,608 (43.6%)	188,452 (45.0%)	25,156 (35.4%)
BMI (kg/m^2^) (mean (SD))	27.4 (4.8)	27.4 (4.7)	27.7 (5.3)
Physical activity (minute/week) (mean (SD))	398.6 (512.4)	410.4 (511.7)	328.8 (511.3)
Loneliness status, loneliness, *n* (%)	22,949 (4.7%)	15,490 (3.7%)	7459 (10.5%)

^a^Positive values of the index will indicate areas with high material deprivation, whereas those with negative values will indicate relative affluence

The numbers and percentages reflect values after multiple imputation for missing covariates

*BMI*, body mass index

### Associations between social isolation and incident diseases and mortality

**Fig. 2:**
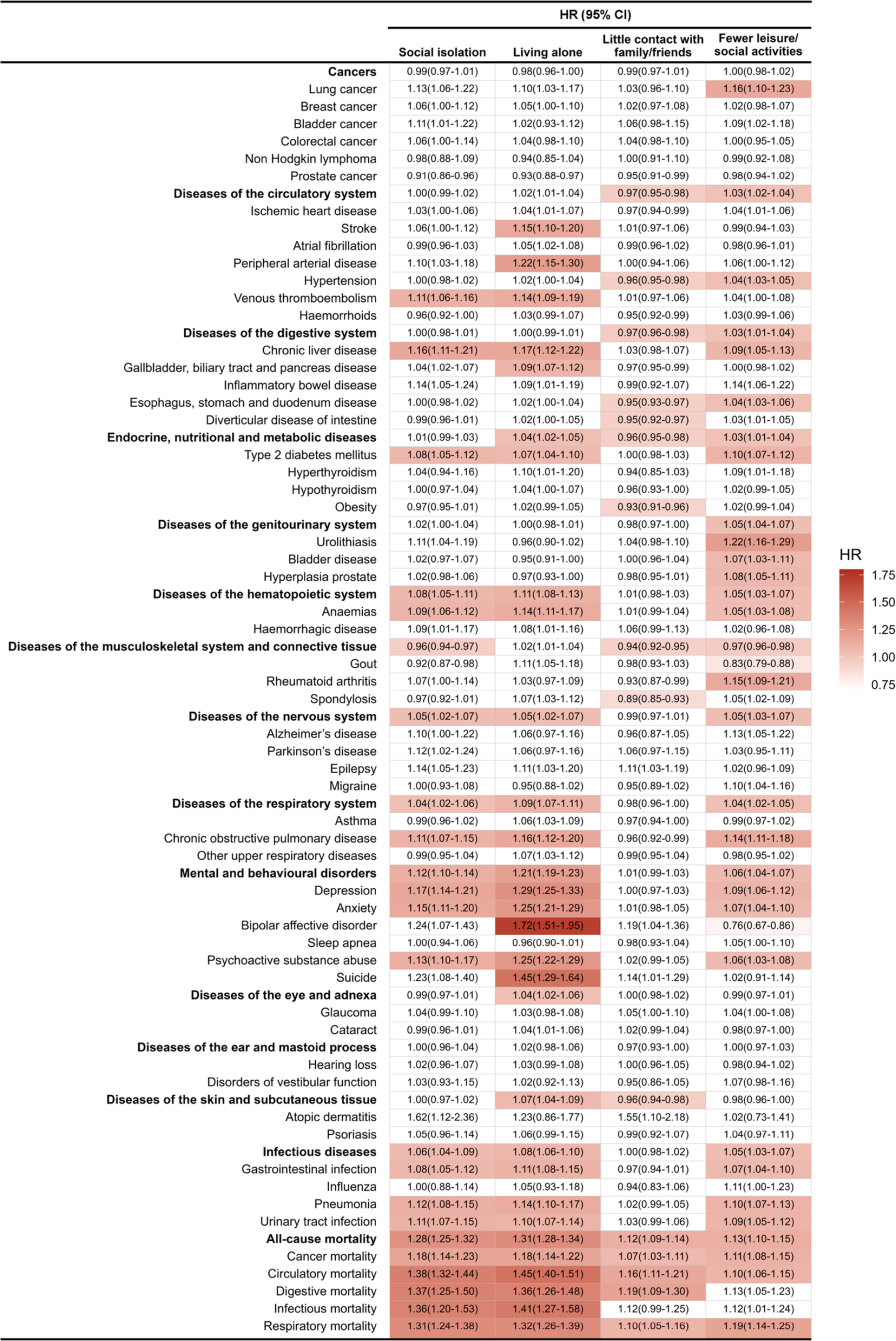
Associations of social isolation and 3 items with diseases among participants from the UK Biobank. HR was adjusted for age, sex, ethnicity, assessment centres, Townsend Deprivation Index, education level, smoking status, alcohol intake frequency, BMI, physical activity, and loneliness status. BMI, body mass index; CI, confidence interval; HR, hazard ratio

The associations of social isolation with the incidence of various disease categories and individual diseases are shown in Fig. 2 and Additional file 2: Table S10. With a median follow-up of 12.5 years, social isolation was significantly associated with 7 out of 15 disease and mortality categories, including increased risks in 6 categories and a modestly reduced risk in 1 category [adjusted hazard ratio (aHR) range, 0.96–1.28]. It was also associated with increased risks for 11 out of 53 individual diseases (aHR range, 1.08–1.17). Furthermore, social isolation was linked to higher mortality rates across all 5 disease-specific categories (aHR range, 1.18–1.38), after adjustment for age, sex, ethnicity, assessment centres, Townsend Deprivation Index, education level, smoking status, alcohol intake frequency, BMI, physical activity, and loneliness status. These associations remained significant after Bonferroni correction for multiple comparisons. Specifically, living alone was associated with increased risks for 9 out of 15 disease and mortality categories (aHR range, 1.04–1.31) and for 21 out of 58 individual diseases and disease-specific mortalities (aHR range, 1.07–1.72). Engaging in fewer leisure or social activities was associated with increased risks for 11 out of 15 categories (aHR range, 0.97–1.13) and for 22 out of 58 individual diseases and disease-specific mortalities (aHR range, 0.76–1.22). In contrast, having little contact with family or friends was associated with a lower number of disease outcomes.

In the analysis of social isolation in relation to fatal and non-fatal diseases, social isolation was associated with a significantly increased risk of several fatal conditions, including genitourinary diseases (aHR, 1.83; 95% CI, 1.31–2.55), circulatory diseases (aHR, 1.75; 95% CI, 1.56–1.96), and respiratory diseases (aHR, 1.47; 95% CI, 1.26–1.71). For non-fatal conditions, higher risks were observed for mental disorders (aHR, 1.12; 95% CI, 1.10–1.14), haematopoietic diseases (aHR, 1.08; 95% CI, 1.05–1.11), infectious diseases (aHR, 1.06; 95% CI, 1.04–1.09), and nervous system diseases (aHR, 1.05; 95% CI, 1.02–1.07). In contrast, social isolation was associated with a slightly decreased risk of non-fatal musculoskeletal diseases (aHR, 0.96; 95% CI, 0.94–0.97). Detailed results are presented in Additional file 2: Tables S11-S12.

### Sensitivity and subgroup analyses

The major results were generally robust across various sensitivity analyses, including repeating the analyses after excluding participants with any missing covariate data (Additional file 2: Table S13), excluding events occurring within the first 2 years (Additional file 2: Table S14), calculating Fine-Gray subdistribution hazards (Additional file 2: Table S15) and performing PSM (Additional file 2: Table S16), conducting NCO analyses (Additional file 2: Table S17), and applying IPW (Additional file 2: Table S18). The associations between social isolation and incident diseases were generally consistent across subgroups stratified by age (< 60 years or ≥ 60 years), sex (male or female) and loneliness status (no loneliness or loneliness) (Additional file 2: Tables S19-S21).

### Associations of genetic liability to social isolation with diseases

**Fig. 3:**
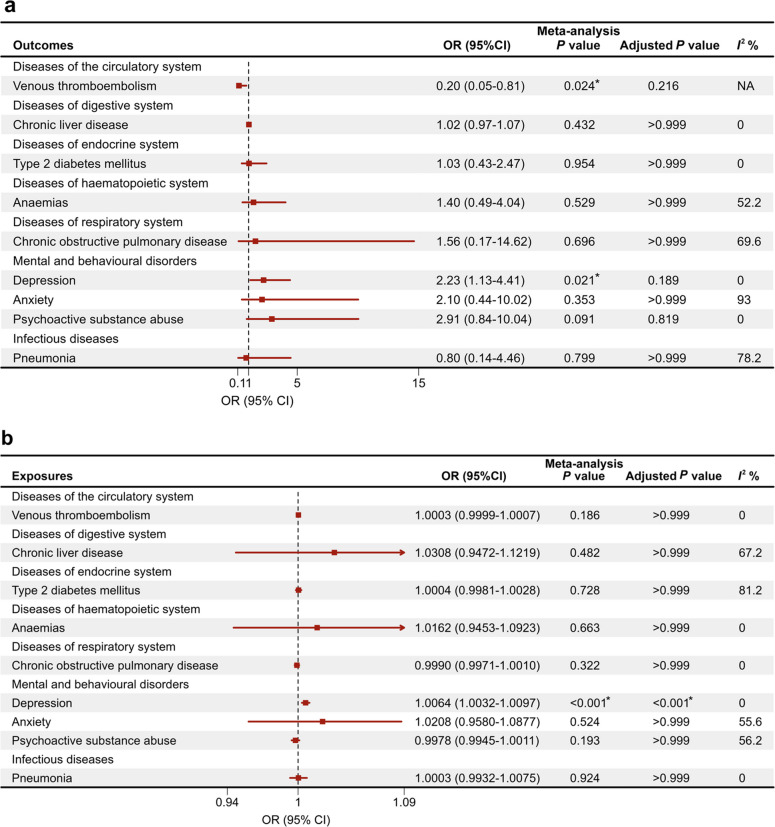
Causal associations between social isolation and diseases using the MRE-IVW method. **a** Causal effects of social isolation on diseases. **b** Causal effects of diseases on social isolation. Adjusted *P* value refers to the *P *value after Bonferroni correction. *I*^2^ values <25%, 25-75% and >75% were considered to indicate low, moderate and high heterogeneity, respectively. *P < 0.05. CI, confidence interval; OR, odd ratio

After conducting a multivariable GWAS with a significance threshold of *P* ≤ 5 × 10^−6^, 13 SNPs of the latent social isolation factor were identified. The meta-analysis of two-sample MR showed potential absence of genetically causal associations between social isolation and 9 individual diseases screened with available genetic sources after Bonferroni correction, including venous thromboembolism, chronic liver disease, type 2 diabetes mellitus (T2DM), anaemias, chronic obstructive pulmonary disease (COPD), depression, anxiety, psychoactive substance abuse, and pneumonia (Fig. 3a). In the other MR direction, after Bonferroni correction, no evidence of a causal effect of any individual disease on social isolation was observed, except for depression [odds ratio (OR), 1.0064; 95% CI, 1.0032–1.0097] (Fig. 3b). The results remained robust in the MR sensitivity analyses. The MR-Egger intercept analyses found no evidence of potential horizontal pleiotropy (Additional file 2: Tables S22-S23). Similarly, no outliers were detected in MR-PRESSO analyses (Additional file 2: Tables S22-S23). The MR meta-analysis (Additional file 1: Figure S2) and sensitivity analyses (Additional file 2: Tables S24-S25) of the three social isolation components also consistently showed limited evidence supporting causal effects in both directions. Furthermore, the MR meta-analysis (Additional file 1: Figure S3) and sensitivity analyses (Additional file 2: Table S26) using the social isolation SNPs identified by Socrates et al. similarly indicated an absence of supportive evidence for causal effects.

### Omics-based machine learning andfunctional pathway enrichment

After adjusting for age, sex, ethnicity, assessment centres, Townsend Deprivation Index, education level, smoking status, alcohol intake frequency, BMI, physical activity, and loneliness status, multiple proteomic and metabolomic markers were associated with a range of disease outcomes, including chronic liver disease, T2DM, anaemias, COPD, mental disorders, pneumonia, urinary tract infections, and all-cause mortality. Additionally, for venous thromboembolism and gastrointestinal infection, only metabolomic markers showed significant associations. Among the proteomic markers, growth differentiation factor 15 (GDF15) emerged as the most predictive across the majority of conditions, whereas among metabolomic markers, glycoprotein acetyls showed the highest predictive value, followed by free cholesterol/total in small low-density lipoprotein (LDL) (%). Selected results of the machine learning analyses, including the top 15 markers with the highest predictive value for each condition, are presented in Additional file 1: Figure S4.

Furthermore, we investigated functional pathways for the proteins with significant explanatory effects for 7 individual diseases and all-cause mortality, which remained significant in the Cox models after Bonferroni correction. For venous thromboembolism and gastrointestinal infection, no proteins showed significant associations, thus enrichment analyses could not be performed. We observed several functional pathways between social isolation and diseases (Additional file 1: Figure S5), including response to stimulus, signaling receptor binding, extracellular region, and cytokine-cytokine receptor interaction.

### Explanatory factors linking social isolation to multiple diseases

**Fig. 4:**
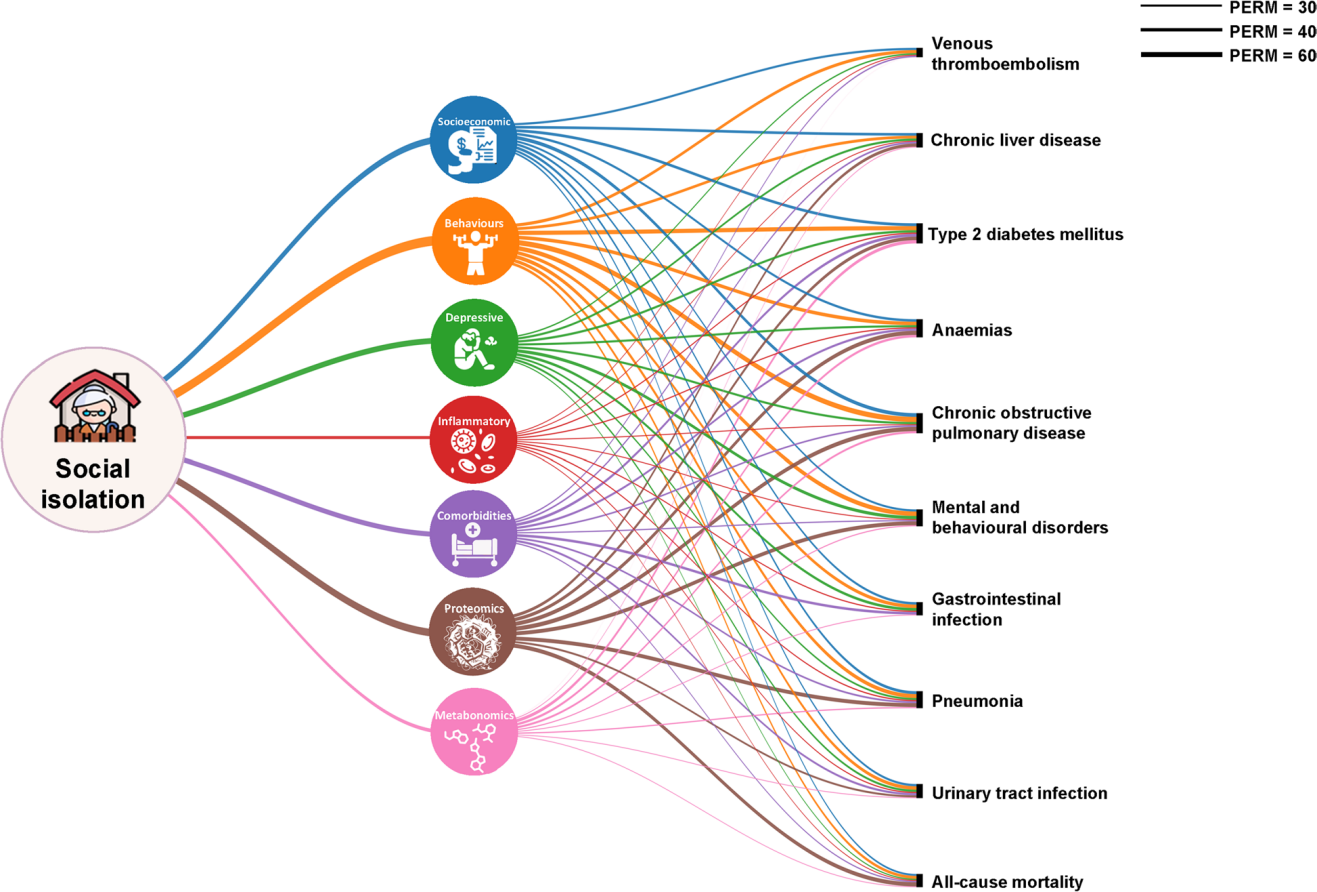
Proportions of associations between social isolation and diseases attributable to different explanatory factors. Minimally adjusted for age, sex, ethnicity, and assessment centres. (1) Socioeconomic factors: education level, Townsend Deprivation Index, and employment; (2) health behaviours: health diet score, smoking status, alcohol intake frequency, sleep duration, and physical activity; (3) depressive symptoms: depression scores measured by Patient Health Questionnaire-2; (4) inflammatory factors: leukocytes, platelets, platelet crit, lymphocytes, monocytes, neutrophils, eosinophils, basophils, C-reactive protein; (5) comorbidities: history of long-standing illness, use of cholesterol-lowering medication, and use of antihypertensive medication; (6) proteomics factors; (7) metabolomics factors. For venous thromboembolism and gastrointestinal infection, no proteins were significantly associated

As shown in Fig. 4 and Additional file 2: Table S27, the associations between social isolation and various diseases were largely explained by other factors. Health behaviours (PERM range, 32%–59%), proteomics signatures (PERM range, 36%–49%), and socioeconomic factors (PERM range, 22%–42%) were the primary contributors to the observed associations between social isolation and diseases. Baseline depression symptoms were stronger explanatory factors for mental and behaviour disorders (PERM, 36%), particularly for depression and anxiety. Metabolomics signatures were more significant in explaining the association with T2DM (PERM, 37%) compared to other diseases. Inflammatory factors (PERM range, 11%–15%) and comorbidities (PERM range, 14%–32%) also played roles in the social isolation-disease associations. Furthermore, in the explanatory analysis focusing on “living alone” (Additional file 2: Table S28). Socioeconomic factors, health behaviours, and proteomic signatures emerged as the main contributors of the associations between living alone and various diseases, consistent with the patterns observed for social isolation.

## Discussion

Leveraging observational, genetic, and proteomic data across 489,741 participants from the UK Biobank, the present study comprehensively investigated associations of social isolation with the risk for 59 health conditions, encompassing 53 specific diseases, 5 cause-specific mortalities, and all-cause mortality. The major finding was that social isolation and each of its items were not associated with most health conditions tested. Instead, social isolation showed strong associations with cause-specific and all-cause mortality, and modest associations with 11 out of the 53 individual diseases considered, such as depression, anxiety, chronic liver diseases, or type 2 diabetes mellitus. Living alone, a cored item of social isolation, demonstrated a comparable pattern as social isolation. However, MR analyses provided little evidence to support a causal effect of genetic liability to social isolation on 9 individual diseases identified. Lastly, plasma proteins involved in the social isolation-health conditions associations were largely related to “response to stimulus”. These proteomic signatures, health behaviours, and socioeconomic factors were predominantly potential explanatory factors linking social isolation with all-cause mortality and diseases.

To our surprise, our comprehensive outcome-wide observational analyses identified only a limited number of significant associations between social isolation and diseases. This finding contrasts markedly with our earlier observation that loneliness, a subjective perception of social disconnection, was linked to a broad spectrum of tested diseases [2]. This discrepancy further reinforces the view that social isolation and loneliness represent two distinct facets of social disconnection [43]. Further, compared with previous studies focusing on single or limited health conditions [6, 12, 44], our study owns strengths in more effectively filtering out false positive findings. This was achieved by employing an outcome-wide approach coupled with rigorous multiple comparison corrections [45]. We also improved upon previous studies by utilizing PSM strategy as a sensitivity analysis to address potential residual confounding, thereby enhancing the reliability of our estimates [46]. Moreover, we firstly identified significant associations of social isolation with several diseases, like anaemias, psychoactive substance abuse, and urinary tract infection. Nevertheless, our findings replicated the significant associations between social isolation and several health conditions like depression [6, 47], anxiety [11], and type 2 diabetes [48].

Benefiting from outcome-wide investigation, we successfully identified mortality as the paramount health risk associated with social isolation, surpassing other health conditions. Extending previous research on mortality [49–51], we established associations between social isolation and 5 cause-specific mortalities, with circulatory and digestive-cause mortalities posing the highest risks. These findings align with previous studies, which similarly found strong associations between social isolation or living alone and mortality, but not non-fatal conditions [9, 13]. Notably, we discovered that the mortality risks attributable to the item “living alone” surpassed those to social isolation or the other two items. This evidence implied that living alone predominantly drives the adverse health effects of social isolation. This may be due to the lack of a companion to assist in seeking immediate healthcare during acute disease events [13]. Thereby, our findings suggested that individuals experiencing social isolation, particularly living alone, are susceptible to mortality and fatal conditions.

Our findings suggested that social isolation exerts independent and distinct health effects compared to loneliness. First, loneliness may represent a more potent psychological stressor than social isolation, because it frequently occurs among individuals at elevated risk for depression and stressful life events [11, 18]. Second, our prior research [14], along with existing evidence, consistently indicated that social isolation shows stronger links to fatal conditions and mortality, whereas loneliness is more frequently associated with mental and metabolic disorders [2, 9, 13, 14, 16]. These findings have suggested that socially isolated individuals may have died of fatal diseases before developing chronic conditions requiring hospitalization. Nevertheless, our results also indicated potential moderating effects of lonely feelings or depressive symptoms on the associations between social isolation and diverse health outcomes.

Our MR analyses found little evidence supporting causal associations of genetic liability to social isolation with 9 diseases identified. In line with a previous study, Socrates et al. [17] found no evidence of causality of genetic liability to social isolation on depression. In contrast, other previous MR studies found causal associations between social isolation and several health conditions, such as T2DM [16], lung cancer and asthma [52]. Notably, all these MR studies employed only single-dimensional indicators of social isolation as instrumental variables, which may not capture the other dimensions of social isolation [53]. To overcome this limitation, we derived an instrumental variable representing the overall components of social isolation by combining SNPs from three items using multivariable common factors GWAS [27]. This newly constructed instrumental variable of social isolation may enhance the statistical power in the MR analysis and minimize the potential bias caused by weak instrumental variable. Furthermore, we advantaged existing MR studies by utilizing non-overlapping samples and applying Bonferroni correction. Taking together, our findings from MR analyses have indicated an absence of causal evidence on associations of social isolation with most diseases.

Several plausible explanations may elucidate the observed associations between social isolation and health conditions. Firstly, previous studies had found that social isolation may foster unfavourable lifestyle behaviours changes, including physical inactivity, inadequate nutrition intake, and substance abuse [54, 55], therefore increasing the risks of mortality and diseases. Consistent with these observations, we found that health behaviours served as an important explanatory factor to the associations between social isolation and all-cause mortality and 9 additional diseases. Secondly, a recent study had reported that the impact of social isolation on mortality varies by country income level and by residential context (e.g., rural or urban) [56]. This is supported by our finding that socioeconomic factors emerge as another major explanatory factor. Finally, living alone also partially accounted for the association between social isolation and mortality, likely through delayed access to timely medical care [9, 50]. Lastly, other unmeasured confounding factors may also explain the observed associations.

Stepping forward, our machine learning and enrichment analyses utilizing high-throughput proteomics data indicate that specific biological processes are potentially associated with social isolation, mortality and adverse health conditions. These enriched processes may serve as phenotype-associated biomarkers of social isolation and disease-related states. We identified that the majority of these proteins are involved in the biological process of “response to stimulus”. Our findings align with previous evidence suggesting that social isolation can function as a chronic social stressor [57], accompanied by heightened stress responses. In accordance with a recent proteomic study [20], we observed cytokine-cytokine receptor interaction and viral protein interaction categories associated with social isolation. Notably, we discovered inflammatory markers, including interleukin-6 (IL-6), interleukin-18 receptor 1 (IL-18R1), and glycoprotein acetyls, as plausible indicators of disease. Our proteomic analyses built upon previous research by considering a broader spectrum of health conditions, encompassing mortality and various additional disease conditions.

This study highlighted that addressing social disconnection alone is insufficient to prevent adverse health outcomes. Instead, as living alone was the strongest indicator of mortality, facilitating quicker healthcare access particularly for people living alone is critical. Moreover, health behaviours, identified as another important explanatory factor, represent a modifiable target through public policies and health strategies. Targeting the key explanatory factors, including unhealthy behaviours, proteomics signatures, socioeconomic factors, depressive symptoms, or comorbidities, may mitigate adverse health conditions among isolated older people [58].

The strengths of this study included its large-scale cohort design, comprehensive evaluation of a wide range of health conditions and potential explanatory factors, and rigorous control of confounding and potential biases through propensity score matching, inverse probability weighting, negative control outcomes, and other sensitivity analyses. Additionally, the study rigorously addressed multiple comparisons and increased statistical power to assess genetically causal relationships using multivariable common factor GWAS.

However, several limitations should be considered. First, most participants in the UK Biobank were relatively healthy [59], potentially leading to an underestimation of disease incidence. Second, social isolation was assessed using three simple self-reported questions from the UK Biobank. Nonetheless, these questions were adapted from validated scales and have been widely used in previous studies [9, 24]. Third, the UK Biobank only assessed in-person contact as an indicator of social isolation, lacking assessments of virtual connections or other aspects of social relationships. Of note, self-reported measures of in-person contact frequency with family and friends are susceptible to recall bias, particularly when online interactions are excluded. Employed participants may have limited time to contact with non-cohabiting families and friends. This may have led to an underestimation of contact frequency, thereby attenuating the estimated associations toward null findings. Fourth, although we employed negative control outcome analyses, PSM and other sensitivity analyses to minimize the impact of confounding factors, the possibility of residual confounding cannot be entirely ruled out in our observational study. Fifth, for certain outcomes and mortality, suitable genetic instruments were unavailable, which limited our ability to investigate their potential causal relationships with social isolation. Sixth, socioeconomic traits generally have low heritability. Although we used high-quality data sources, the genetic instruments derived from the latent factor GWAS may still be susceptible to weak instrument bias, which could have contributed to the null findings. Therefore, a causal relationship between social isolation and the outcomes cannot be definitively ruled out. In addition, reverse causation cannot be fully excluded, whereby subclinical disease may influence inflammatory marker levels. However, we have excluded deaths or first events occurring within the first 2 years, which yielded robust findings. Finally, since the majority of participants in the UK Biobank were of European descent, caution is needed when generalizing our findings to other ethnic groups.

## Conclusions

Although the MR analyses did not provide genetic evidence of causality, our study overall demonstrated that social isolation was significantly associated with increased risks of diverse adverse health outcomes, encompassing 6 mortality outcomes and 11 specific diseases. To enhance overall health in an ageing society facing inevitable social isolation, it is essential to strengthen social ties, ensure timely medical assistance, and address underlying modifiable risk factors.

## Supplementary Information

Additional file 1: Methods S1-S9. Methods S1 - Detailed information on the participant selection. Methods S2 - Detailed information on exposures. Methods S3 - Detailed information on covariates and explanatory factors. Methods S4 - Detailed information on Mendelian randomization. Methods S5 - Detailed information on propensity score matching. Methods S6 - Detailed information on negative control outcome analysis. Methods S7 - Detailed information on inverse probability weighting. Methods S8 - Detailed information on functional enrichment analyses. Methods S9 - Detailed information on machine learning analyses. Figures S1-S5. Figure S1 - Analytical frameworks for proteomic and metabolomic analyses. Figure S2 - Causal associations between items of social isolation and diseases using the MRE-IVW method. Figure S3 - Causal effects of social isolation behavior identified by Socrates et al. on multiple diseases using the MRE-IVW method. Figure S4 - Proteomics and metabolomics analysis between social isolation and multiple diseases. Figure S5 - Enrichment analysis of proteins linking social isolation and multiple diseases.

Additional file 2: Tables S1-S28. Table S1 - Information about exposures, outcomes, covariables, and explanatory factors in UK Biobank. Table S2 - Information regarding the studies and consortia included in MR analyses. Table S3 - Information on SNPs used as instrumental variables for social isolation (n=13). Table S4- Information on SNPs used as instrumental variables for living alone (n=24). Table S5 - Information on SNPs used as instrumental variables for little contact with family or friends (n=109). Table S6 - Information on SNPs used as instrumental variables for fewer leisure/social activities (n=81). Table S7 - Information on SNPs used as instrumental variables for social isolation behavior identified by Socrates et al. (n=17). Table S8 - Definition of outcome measures in the FinnGen consortium in MR analyses. Table S9 - Baseline characteristics of participants after propensity score matching. Table S10 - Associations of social isolation and items with multiple diseases among participants from UK Biobank. Table S11 - Associations between social isolation and multiple fatal or non-fatal diseases among participants from UK Biobank. Table S12 - Associations between items of social isolation and multiple fatal or non-fatal diseases among participants from UK Biobank. Table S13 - Sensitivity analysis of associations of social isolation and items with multiple diseases among participants from UK Biobank with complete variable data. Table S14 - Sensitivity analysis of associations of social isolation and items with multiple diseases among participants from UK Biobank after excluding the events occurring within the first two years. Table S15 - Sensitivity analysis of associations of social isolation and multiple diseases among participants from UK Biobank by using competing risk regression. Table S16 - Sensitivity analysis of associations of social isolation and items with multiple diseases among participants from UK Biobank after propensity score matching. Table S17 - Sensitivity analysis of associations between social isolation and negative control outcome (nonsuppurative otitis media) among participants from UK Biobank. Table S18 - Sensitivity analysis of associations of social isolation and items with multiple diseases among participants from UK Biobank after inverse probability weighting. Table S19 - Subgroup analyses of associations of social isolation and multiple diseases among participants from UK Biobank stratified by age. Table S20 - Subgroup analyses of associations of social isolation and multiple diseases among participants from UK Biobank stratified by sex. Table S21 - Subgroup analyses of associations of social isolation and multiple diseases among participants from UK Biobank stratified by loneliness status. Table S22 - Sensitivity analysis of the causal effects of social isolation on multiple diseases in EBI and FinnGen using multiple Mendelian randomization methods. Table S23 - Sensitivity analysis of the causal effects of multiple diseases on social isolation in EBI and FinnGen using multiple Mendelian randomization methods. Table S24 - Sensitivity analysis of the causal effects of 3 items on multiple diseases in EBI and FinnGen using multiple Mendelian randomization methods. Table S25 - Sensitivity analysis of the causal effects of multiple diseases on 3 items in EBI and FinnGen using multiple Mendelian randomization methods. Table S26 - Sensitivity analysis of the causal effects of social isolation behavior identified by Socrates et al. on multiple diseases in EBI and FinnGen using multiple Mendelian randomization methods. Table S27 - Results of Cox-based mediation analysis (social isolation). Table S28 - Results of Cox-based mediation analysis (living alone).

## Data Availability

Individual-level data from the UK Biobank are not publicly accessible due to institutional policy but can be obtained upon approval of a formal application to the UK Biobank (https://www.ukbiobank.ac.uk/) [60]. The GWAS data used in the MR study were sourced from the UK Biobank, the European Bioinformatics Institute (EBI) and the FinnGen consortium (Round 10). The social isolation data from the UK Biobank are sourced from the IEU Open GWAS project [61–63]. Detailed information and access to the resource are available at https://gwas.mrcieu.ac.uk/. Data from EBI can be accessed at https://www.ebi.ac.uk/gwas/ [64–70]. Details and access to the data from the FinnGen consortium (Round 10) are available at https://www.finngen.fi/en/access_results [71]. The GWAS data for social isolation behavior used in the MR sensitivity analyses were obtained from Socrates et al. [PMID: 38811692] upon request from the corresponding author. Scripts utilized for conducting the analyses can be accessed via GitHub at [72].

## Abbreviations

MR: Mendelian randomization
ICD-10: The International Classification of Diseases-10th Revision
BMI: Body mass index
GWAS: Genome-wide association studies
SNPs: Single nucleotide polymorphisms
LDSC: Linkage disequilibrium score regression
MRE-IVW: The random-effects multiplicative inverse variance-weighted
MR-PRESSO: The MR pleiotropy Residual Sum and Outlier
UKB-PPP: The UK Biobank Pharma Proteomics Project
SD: Standard deviation
IQR: Interquartile range
HR: Hazard ratio
CI: Confidence interval
PSM: Propensity score matching
NCO: Negative control outcome
IPW: Inverse probability weighting
XGBoost: EXtreme Gradient Boosting
SHAP: Shapley Additive Explanations
PERM: Percentage of excess risk mediated
aHR: Adjusted hazard ratio
T2DM: Type 2 diabetes mellitus
COPD: Chronic obstructive pulmonary disease
OR: Odds ratio
GDF15: Growth differentiation factor 15
LDL: Low-density lipoprotein
IL-6: Interleukin-6
IL-18R1: Interleukin-18 receptor 1
