# Supplementary material for: Social isolation and 59 common health conditions: insights from observational and genetics analyses
Source: BMC Med. 2026 Mar 23;24:282. doi: 10.1186/s12916-026-04784-1 (PMC13134139; doi:10.1186/s12916-026-04784-1)
Supplement: Supplementary file 1 — Additional file 1: Methods S1-S9. Methods S1 - Detailed information on the participant selection. Methods S2 - Detailed information on exposures. Methods S3 - Detailed information on covariates and explanatory factors. Methods S4 - Detailed information on Mendelian randomization. Methods S5 - Detailed information on propensity score matching. Methods S6 - Detailed information on negative control outcome analysis. Methods S7 - Detailed information on inverse probability weighting. Methods S8 - Detailed information on functional enrichment analyses. Methods S9 - Detailed information on machine learning analyses. Figures S1-S5. Figure S1 - Analytical frameworks for proteomic and metabolomic analyses. Figure S2 - Causal associations between items of social isolation and diseases using the MRE-IVW method. Figure S3 - Causal effects of social isolation behavior identified by Socrates et al. on multiple diseases using the MRE-IVW method. Figure S4 - Proteomics and metabolomics analysis between social isolation and multiple diseases. Figure S5 - Enrichment analysis of proteins linking social isolation and multiple diseases. [file 12916_2026_4784_MOESM1_ESM.docx]

**Additional file 1**

**Title: Social isolation and 59 common health conditions: insights from observational and genetics analyses**

| Methods S1 | Detailed information on the participant selection | 2 |
| --- | --- | --- |
| Methods S2 | Detailed information on exposures | 3 |
| Methods S3 | Detailed information on covariates and explanatory factors | 4 |
| Methods S4 | Detailed information on Mendelian randomization | 5 |
| Methods S5 | Detailed information on propensity score matching | 6 |
| Methods S6 | Detailed information on negative control outcome analysis | 7 |
| Methods S7 | Detailed information on inverse probability weighting | 8 |
| Methods S8 | Detailed information on functional enrichment analyses | 8 |
| Methods S9 | Detailed information on machine learning analyses | 9 |
| Figure S1 | Analytical frameworks for proteomic and metabolomic analyses | 11 |
| Figure S2 | Causal associations between items of social isolation and diseases using the MRE-IVW method | 12 |
| Figure S3 | Causal effects of social isolation behavior identified by Socrates et al. on multiple diseases using the MRE-IVW method | 14 |
| Figure S4 | Proteomics and metabolomics analysis between social isolation and multiple diseases | 15 |
| Figure S5 | Enrichment analysis of proteins linking social isolation and multiple diseases | 18 |

**Methods S1. Detailed information on the participant selection**

In the UK Biobank study, at baseline, a subset of participants (n = 502,505) was invited to complete the touchscreen questionnaires between 2006 and 2010. Participants with missing data on “living alone”, “little contact with friends or family” or “fewer leisure/social activities” were excluded (n = 12,622). Additionally, participants who had withdrawn from the study (n = 142) were excluded, resulting in a final study sample of 489,741 participants with valid and complete responses regarding social isolation. Furthermore, participants with a diagnosis of each disease at baseline were excluded from the analysis.

**Methods S2. Detailed information on exposures**

| **Social isolation** | | | | | |
| --- | --- | --- | --- | --- | --- |
| Questions | Field ID | Answers | Score of each question | Definition of each item | Total score (range 0–3) |
| (1) Including yourself, how many people are living together in your household? | 709 | Number in household/Do not know/Prefer not to answer | 1 = The answer was one person^†^  0 = Other answers | **Living alone** was defined by question (1).  1 = Living alone  0 = Not living alone | Total score was the sum of the scores from the three questions.  2 or 3 = Socially isolated  0 or 1 = Not isolated |
| (2) How often do you visit friends or family or have them visit you? | 1031 | Almost daily/2–4 times a week/About once a week/About once a month/Once every few months/Never or almost never/No friends or family outside household/Do not know/Prefer not to answer | 1 = About once a month/Once every few months/Never or almost never/No friends or family outside the household^‡^  0 = Almost daily/2–4 times a week/About once a week | **Little contact with family or friends** was defined by question (2).  1 = Little contact with family or friends  0 = More contact with family or friends |  |
| (3) Which of the following do you attend once a week or more often? | 6160 | Sports club or gym/Pub or social club/Religious group/Adult education class/Other group activity/None of the above/Prefer not to answer | 1 = None of the above^*^  0 = Sports club or gym/Pub or social club/Religious group/Adult education class/Other group activity | **Fewer leisure/social activities** was defined by question (3).  1 = Fewer leisure/social activities  0 = More leisure/social activities |  |

^†^If the answer was < 1, > 100, “Do not know” or “Prefer not to answer”, then we excluded it.

^‡^If the answer was “Do not know” or “Prefer not to answer”, then we excluded it.

^*^If the answer was “Do not know” or “Prefer not to answer”, then we excluded it.

**Methods S3. Detailed information on covariates and** **explanatory factors**

In the UK Biobank, we utilized the self-reported questionnaires, physical-measured variables, and medical history to assess potential confounders. Age (field ID 21003) was calculated based on date of birth. Sex (Female, Male; field ID 31), ethnicity (White, Others; field ID 21000), assessment center (England, Scotland, Wales; field ID 54), and Townsend Deprivation Index (continuous; a higher score indicates a higher degree of deprivation; field ID 189), derived from participants’ postcode, were obtained from self-reported questionnaires. Covariate data collected through touchscreen questions at baseline included: education level (college or university degree, non-college or university degree; field ID 6138), healthy diet score (continuous, 0-5 points; field ID 1289, 1299, 1309, 1319, 1329, 1339, 1369, 1379, 1389, and 1349), current employment status (employed, unemployed; field ID 6142), smoking status (never, previous, current; field ID 20116), alcohol intake frequency (not current, two or less times a week, three or more times a week; field ID 1558), body mass index (BMI, continuous, in kg/m2; field ID 21001), sleep duration (continuous, hours/day; field ID, 1160), physical activity (continuous, minutes/week; field ID 22038, 22039), and loneliness status (loneliness, no loneliness; field ID 2020, 2110). Biochemical markers assessed from blood samples included leukocytes (continuous, 10^9^ cells/Litre; field ID, 30000), platelets (continuous, 10^9^ cells/Litre; field ID, 30080), platelet crit (continuous, percent; field ID, 30090), lymphocytes (continuous, 10^9^ cells/Litre; field ID, 30120), monocytes (continuous, 10^9^ cells/Litre; field ID, 30130), neutrophils (continuous, 109 cells/Litre; field ID, 30140), eosinophils (continuous, 10^9^ cells/Litre; field ID, 30150), basophils (continuous, 10^9^ cells/Litre; field ID, 30160), and C-reactive protein (continuous, mg/L; field ID, 30710).

Specifically, the healthy diet score was calculated based on the following dietary factors: vegetable intake at least four tablespoons each day (median); fruits intake at least three pieces each day (median); fish intake at least twice each week (median); unprocessed red meat intake no more than twice each week (median); and processed meat intake no more than two each week (median). One point was assigned for each favorable dietary factor, resulting in a total diet score ranging from 0 to 5.

Information on longstanding illnesses, disability, or infirmity was collected through touchscreen questions at baseline (field ID 2188). Depressive symptoms were assessed using two items from the Patient Health Questionnaire-2 (PHQ-2): the frequency of depressed mood (field ID 2050) and disinterest or absence of enthusiasm (field ID 2060) over the past 2 weeks. For each item, if participants answered, “not at all”, “several days”, “more than half the days”, or “nearly every day”, they were coded as 0, 1, 2, and 3, respectively. The total depression score, ranging from 0 to 6, was calculated by summing the scores of the two items.

**Methods S4. Detailed information on Mendelian randomization**

In our two-sample Mendelian randomization (MR) study, genetic instrumental variables for all items of social isolation (“number in household”, “frequency of friend/family visits”, and “leisure/social activities: none of the above”) were derived from genome-wide association studies (GWAS) conducted in the UK Biobank population (GWAS ID: ukb-b-5445, ukb-b-5379, and ukb-b-5076). The selection criteria for genetic instruments in our study were as follows: 1) Genome-wide significance (*P* < 5×10^-6^); 2) Linkage disequilibrium among SNPs associated with each exposure was calculated using the PLINK clumping method, based on the 1000 Genomes European panel. SNPs with r² > 0.001 and a clumping distance <10,000 kilobases (kb) were excluded. Instrumental variables for disease outcomes were obtained from the FinnGen consortium (Round 10) (<https://www.finngen.fi/en/access_results>) and the European Bioinformatics Institute (EBI) (<https://www.ebi.ac.uk/gwas/downloads>). Detailed GWAS information for social isolation and the diseases is provided in Tables S22-28.

For MR sensitivity analyses, we used genetic variants associated with social isolation behavior identified by Socrates et al., who conducted a GWAS on a composite social isolation phenotype based on three continuous measures. In our analysis, SNPs were selected based on genome-wide significance (*P* < 5×10⁻⁸) and linkage disequilibrium calculated using the PLINK clumping method with the 1000 Genomes European reference panel. SNPs with r² > 0.001 or a clumping distance <10,000 kilobases (kb) were excluded. To ensure the validity of the instruments, SNPs showing nominal associations with the disease outcomes (*P* < 0.05) were further removed. The strength of each instrument was assessed using F-statistics, with values below 10 indicating potential weak instruments. Causal estimates were obtained using the random-effects multiplicative inverse variance-weighted (MRE-IVW) method. Additional sensitivity analyses were conducted using the weighted median and MR-Egger methods to provide alternative causal estimates, while horizontal pleiotropy and heterogeneity across SNPs were evaluated with MR-PRESSO and Cochran’s Q test Detailed GWAS information for social isolation and disease outcomes is provided in Table S22-28.

**Methods S5. Detailed information on propensity score matching**

To reduce selection bias arising from differences in individual characteristics, a matched control was randomly selected for each participant with social isolation from participants without social isolation by using the propensity score method, which accounted for age, sex, ethnicity, assessment centres, Townsend Deprivation Index, education level, smoking status, alcohol intake frequency, BMI, physical activity, loneliness status.

**Methods S6. Detailed information on negative control outcome analysis**

Negative control analysis was performed to address potential bias arising from unmeasured confounding factors. The negative control analysis was based on two reasonable previous assumptions: (1) zero causality, meaning that the exposures or outcomes of the control should not be causally related to the exposures or outcomes of interest; and (2) U-comparability, meaning that the exposures or outcomes of the control should share a similar confounding structure with those of interest. The negative control outcome (NCO) analysis relies on an NCO variable that is not affected by the exposure but shares the same unmeasured confounders that may bias the exposure-outcome association of interest. Such an NCO is therefore considered “U-comparable” to the outcome. If, after adjusting for all measured confounders, a residual association is still observed between the exposure and the NCO, this indicates the presence of unmeasured confounding. Conversely, a null association suggests that unmeasured confounding is unlikely to meaningfully bias the primary results. Based on these principles, “nonsuppurative otitis media” was selected as the NCO, as no evidence supports an association between this condition and social isolation, and it is likely to be U-comparable with the other disease outcomes.

**Methods S7. Detailed information on inverse probability weighting**

Inverse probability weighting was conducted to reduce imbalance in measured confounders and to address selection bias arising from the non-random, health-conscious volunteer participation characteristic of the UK Biobank, which can generate systematic differences between social isolation groups. Propensity scores were estimated using logistic regression models, with social isolation as the dependent variable and multiple baseline covariates as independent variables. IPW was implemented using the ipw package (version 1.2.1.1), specifying a logit link and unstabilized weights (numerator = 1).

**Methods S8. Detailed information on functional enrichment analyses**

We selected proteins significantly associated with diseases and performed protein functional enrichment analysis using the STRING platform (<https://string-db.org/>). To provide a comprehensive functional overview, we incorporated four widely used ontology databases: GO Biological Process (GOBP), GO Molecular Function (GOMF), GO Cellular Component (GOCC), and the Kyoto Encyclopedia of Genes and Genomes (KEGG). GOBP describes the biological processes and pathways in which proteins participate, GOMF captures their molecular activities such as binding or catalytic functions, and GOCC identifies the cellular or subcellular locations where these proteins are active. KEGG provides pathway-level insights by mapping proteins to metabolic, signaling, or disease-related pathways. These databases offer complementary information—what the proteins do, where they act, how they function, and which pathways they influence. For each database, we listed the top five enriched terms with the highest enrichment counts.

**Methods S9. Detailed information on machine learning analyses**

Machine learning analyses were conducted using Python (version 3.8) to evaluate the predictive contributions of significant omics signatures. Protein and metabolic features were used to train an XGBoost model with a maximum tree depth of 3, a learning rate of 0.3, 100 boosting iterations, and a binary logistic objective function. The dataset was randomly split into a training set (70%) and a test set (30%) with a fixed random seed to ensure reproducibility. Model interpretation was performed using Shapley Additive explanations (SHAP), and the mean absolute SHAP value across all test samples was used to rank feature importance. The distribution of SHAP values across individual samples was depicted using beeswarm plots. The top 15 proteins or metabolic markers with the highest predictive values were reported. For data analysis and visualization, the following python packages were used: pandas (v.2.0.3), numpy (v.1.24.4), matplotlib (v.3.7.5), shap (v.0.44.1), xgboost (v.2.1.4).

**Figure S1.** **Analytical frameworks for proteomic and metabolomic analyses**

When MR supported a genetic causal association, individual proteins and metabolites were evaluated as potential downstream mediators in a sequential model; when MR analyses yielded null findings, proteomic and metabolomic signals were interpreted as biomarkers that may act as mediators, confounders, or colliders, operating in a parallel model.

**Figure S2. Causal associations between items of social isolation and diseases using the MRE-IVW method. a,** Causal effects of items on diseases. **b,** Causal effects of diseases on items. Adjusted *P* value refers to the *P* value after Bonferroni correction. *I*^2^ values <25%, 25 - 75% and >75% were considered to indicate low, moderate and high heterogeneity, respectively. **P* < 0.05.

CI, confidence interval; OR, odd ratio.

**Figure S3. Causal effects of social isolation behavior identified by Socrates et al. on multiple diseases using the MRE-IVW method.** Adjusted *P* value refers to the *P* value after Bonferroni correction. *I*^2^ values <25%, 25 - 75% and >75% were considered to indicate low, moderate and high heterogeneity, respectively.

CI, confidence interval; OR, odd ratio.

**Figure S4. Proteomics and metabolomics analysis between social isolation and multiple diseases.** **a**, Machine learning results of proteomics for social isolation and multiple diseases. **b**, Machine learning results of metabolomics for social isolation and multiple diseases.

DHA, docosahexaenoic acid; Ile, isoleucine; Leu, leucine; Total BCAAs, total concentration of branched-chain amino acids (Leucine + Isoleucine + Valine); Val, valine.

SHAP values indicate the direction and magnitude of the feature’s contribution to the model prediction, with higher absolute values reflecting stronger influence. The color scale represents the corresponding feature values used by the model (blue = lower values; red = higher values). Features are ordered by their mean absolute SHAP values across all samples, with those at the top having the greatest overall predictive importance. For venous thromboembolism and gastrointestinal infection, no proteins were significantly associated.

**Figure S5. Enrichment analysis of proteins linking social isolation and multiple diseases.** The top five significantly enriched pathways from each database are displayed, with the $\mathcal{x}$ axis representing -log_10_ *P* FDR. For venous thromboembolism and gastrointestinal infection, no proteins were significantly associated.
